# Marine Non-Glycosaminoglycan Sulfated Glycans as Potential Pharmaceuticals

**DOI:** 10.3390/ph8040848

**Published:** 2015-12-10

**Authors:** Vitor H. Pomin

**Affiliations:** Program of Glycobiology, Institute of Medical Biochemistry Leopoldo de Meis, University Hospital Clementino Fraga Filho, Federal University of Rio de Janeiro, Rio de Janeiro, RJ 21941-913, Brazil; E-Mail: pominvh@bioqmed.ufrj.br or vhpomin@gmail.com; Tel.: +55-21-3938-2939; Fax: +55-21-3938-2090

**Keywords:** angiogenesis, cancer, coagulation, inflammation, marine glycans, microbial infection, sulfated fucans, sulfated galactans, thrombosis

## Abstract

Sulfated fucans (SFs) and sulfated galactans (SGs) are currently the marine non-glycosaminoglycan (GAG) sulfated glycans most studied in glycomics. These compounds exhibit therapeutic effects in several pathophysiological systems such as blood coagulation, thrombosis, neovascularization, cancer, inflammation, and microbial infections. As analogs of the largely employed GAGs and due to some limitations of the GAG-based therapies, SFs and SGs comprise new carbohydrate-based therapeutics available for clinical studies. Here, the principal structural features and the major mechanisms of action of the SFs and SGs in the above-mentioned pathophysiological systems are presented. Discussion is also given on the current challenges and the future perspectives in drug development of these marine glycans.

## 1. Introduction

Glycosaminoglycans (GAGs) are the most therapeutically explored carbohydrates of the pharmaceutical market [1,2]. They belong to a class of sugars named sulfated glycans. Among various types, the commonly used GAGs in medicine are heparin, chondroitin sulfate and keratan sulfate. Heparin is a potent anticoagulant and antithrombotic agent [3,4]. Chondroitin sulfated is used in cases of osteoarthritis, osteoarthrosis and sometimes osteoporosis [5,6,7]. Keratan sulfated is explored as a functional ingredient of eye-drops in therapies of corneal dysfunctions [8,9]. Despite the large application of these GAGs in medicine, they still present some downsides. For example, heparin-based treatments offer serious bleeding and hemorrhage risks [10,11,12]. Chondroitin sulfate-based formulation destined to oral administration was detected contaminated with another GAG type due to rough large-scale preparation methods [7]. Keratan sulfate is only clinically active in corneal dystrophies characterized by structural defects on keratan sulfate-containing proteoglycans [13]. This gives a restricted medical application to keratan sulfate.

However, non-GAG sulfated glycans endowed also with medical actions are becoming more and more available as the glycomics evolves. The marine sulfated fucans (SFs) and sulfated galactans (SGs) are examples of these glycans [14,15,16]. Two reasons to explain the growing interest of these molecules are as follows. (i) The chemical structures of the relatively new marine sulfated glycans are very unique and distinct if compared to GAG structures. Although SFs and SGs bear sulfation and are sometimes composed of disaccharide repeating units like GAGs, the marine sulfated glycans (especially those extracted from invertebrates and red algae) are much more homogeneous and regular in terms of backbone composition and sulfation patterns than GAGs [2,14,15,16]. (ii) Although resembling the mechanisms of action of the mammalian-derived GAGs of medicine, SFs and SGs can exhibit additional or slightly distinct effects. The serpin-independent anticoagulant action of some marine sulfated glycans is an example of additional effect since this mechanism is non-existent in heparin-based treatments [17,18,19]. The pronounced heparin cofactor II (HCII)-driven serpin-dependent anticoagulant activity of some marine SFs and SGs [20,21], as opposed to the main antithrombin (AT)-driven anticoagulant mechanism of action of heparin [21,22], is an example of slightly different effect since both sulfated glycans present serpin-dependent activities although acting on distinct primary serpin-targets (AT or HCII). These additional and slightly distinct effects of the marine sulfated glycans can be considered advantageous factors in the development of alternative anticoagulants, especially in cases in which heparin could be less effective or inactive. Examples of these cases are, respectively, heparin preparations with low concentration of the AT high-affinity pentasaccharide [23], and patients suffering from congenital or acquired disorders characterized by decreased levels of circulating serpins [21,24,25]. In these deficiencies, anticoagulant/antithrombotic therapies unrelated to serpin activities would be better in order to achieve the desired anticoagulant outcome. Moreover, as opposed to heparin which presents bleeding risks as already mentioned, certain SFs and SGs do not exhibit this side-effect [19,26].

This report aims therefore at discussing the prospects of the marine SFs and SGs as potential pharmaceuticals of the future medicine, not only in coagulation and thrombosis but also in other systems in which these molecules have been reported to be effective. The additional systems are angiogenesis, cancer, inflammation and microbial infections. Therefore, molecular details of major mechanisms of action of SFs and SGs in these pathophysiological systems are highlighted here in order to offer a comprehensive overview of their medical properties. Final discussion focuses on the current obstacles and expected challenges to implement these marine sulfated glycans in the next generation of potential carbohydrate-based therapeutics of the global market.

## 2. Structure

**Figure 1:**
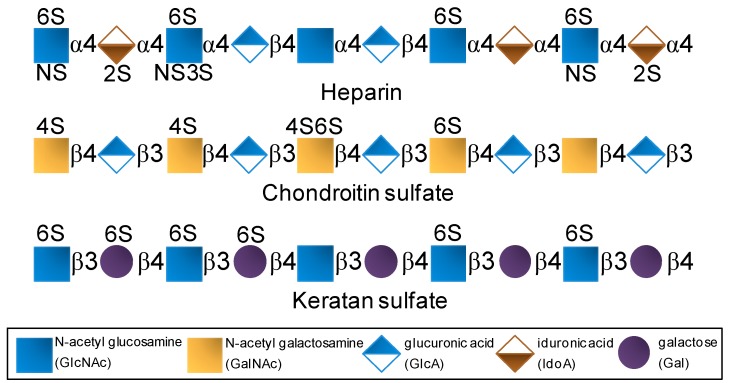
Structural representation of the most explored therapeutic glycosaminoglycans of the global pharmaceutical market.

GAGs are composed of disaccharide repeating building blocks composed of alternating hexosamine and hexuronic acid or galactose (Gal) units (Figure 1). The hexosamine can be glucose-based (glucosamine, GlcN) as seen in heparin and keratan sulfate or galactose-based (galactosamine, GalN) as seen in chondroitin sulfate. The hexuronic acids can be glucuronic acid (GlcA) or its C5-epimer iduronic acid (IdoA). GlcA can be found in chondroitin sulfate and in heparin although at lower proportions since heparin is largely composed of IdoA. Besides variation in glycosidic linkage type, but never different from 3- or 4-positions, GAGs also vary in terms of substitutions at *N*- and/or *O*-positions (Figure 1). For instance, while heparin is largely *N*-sulfated, *O*-sulfated frequently at positions C6 and rarely at C3 of the composing GlcN units with additional sulfation at the C2 position of the IdoA units [27,28,29], chondroitin sulfate bears sulfation mostly at positions C4 and/or C6 of its *N*-acetyl GalN (GalNAc) units (Figure 1) [30,31]. In keratan sulfate, sulfation can occur at C6 positions of either unit (Gal or GlcNAc) but more often at the amino sugar (Figure 1) [7,8,13]. Although composed of disaccharide units and heavily sulfated, GAGs are very complex and heterogeneous in terms of structure. Sulfation is not absolutely regular in any GAG type. It varies within the disaccharide building blocks. The levels of epimerization in GlcA to IdoA in heparin also vary considerably throughout the composing disaccharides.

**Figure 2:**
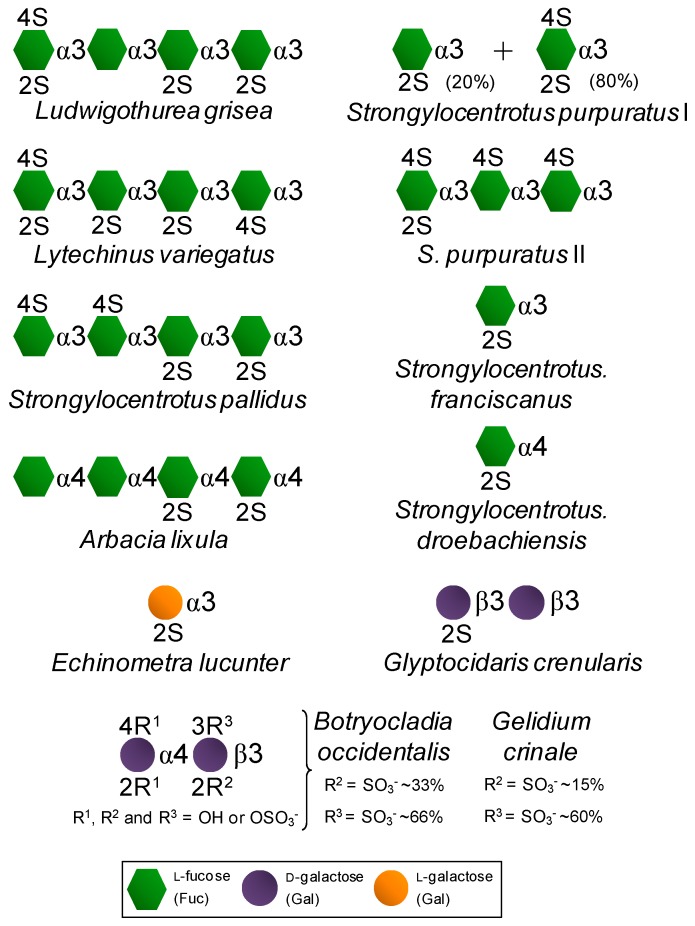
Structural representation of the marine, invertebrate and red algal, SFs and SGs of well-defined chemical structures. All species are sea urchins, except the first one which is a sea cucumber and the last two which are red algae.

On the other hand, certain SFs and SGs, especially those extracted from red algae and invertebrate animals, are fairly homogeneous in terms of monosaccharide composition/distribution and very regular in terms of sulfation patterns (Figure 2) if compared to the therapeutic GAGs (Figure 1). While red algae express only SGs composed of disaccharide repeating units (Figure 2) like GAG molecules (Figure 1), SFs and SGs of regular chemical structures are composed of oligosaccharide (mono to tetrasaccharides) repeating units. These molecules can be extracted from sea urchins, sea cucumbers and ascidians (Figure 2) [14,15,16]. While in sea urchins the SFs and SGs comprise extracellular matrix (ECM) components of the egg jelly coat in female gametes of this echinoderm, in sea cucumbers and ascidians they occur as ECM components of their body walls. While SFs are polymers solely composed of fucopyranose (Fuc) units, SGs are made up primarily of Gal units (Figure 2). In brown algae, only SFs have been reported, however within a more heterogeneous backbone composition [32,33,34,35]. These brown algal SFs are best known as fucoidans and other monosaccharide types have been detected [32,33,34,35]. SGs are the major sulfated glycan in green algae [14,19,36,37]. Although more homogeneous than brown algal fucoidans, green algal SGs are still more heterogeneous than the disaccharide-composed SGs of red algae [14,19,36,37]. Regardless of the phylogenetic classification, sulfation patterns are likely to vary among different species.

In summary, SFs or SGs isolated from invertebrate animals and red algae are very homogeneous in terms of backbone constitution and sulfation patterns (Figure 2). This makes these particular marine sulfated glycans very useful molecular tools to be investigated in medicine specially for establishing advanced structure-activity relationship (SAR). These advanced SAR analyses are relevant in drug discovery since it helps to unveil (i) the underlying mechanisms of action of their medical properties in the different pathophysiological systems, and (ii) the best structural motifs responsible for the highest clinical responses. Although these two aspects can be safely investigated in GAG studies, it is much more difficult to design SAR analyses on GAGs than on the marine SFs and SGs of well-defined chemical structures. Table 1 highlights the major structural aspects of both sulfated glycan types discussed in this report. Outcomes, advantageous as well disadvantageous aspects regarding their medical potentials of each molecular type are also highlighted.

**Table 1:** Comparative structural and medical aspects of glycosaminoglycans (GAGs) and marine sulfated glycans of well-defined chemical structures.

Polysaccharide Type	Molecular Type	Structural Units	Overall Aspects	Medical Benefits
GAGs	Heparin	IdoA2S + GlcNS6S	As the most negatively charged biomacromolecule in nature, it interacts and regulates various protein types.	Potent anticoagulant and antithrombotic agent [3,4].
	Chondroitin sulfate	GlcA + GalNAc(4S and/or 6S)	The most abundant GAG of the body and of connective (cartilage) tissues.	Used in cases of osteoarthritis, osteoarthrosis and sometimes osteoporosis [5,6,7].
	Keratan sulfate	Gal + GlcNAc (6S at both units but more often at GlcNAc)	Highly abundant in cornea. Related with the proper visual functions.	Explored as functional ingredient in eye-drops [8,9].
Marine glycans	SFs	Fuc (2S and/or 4S)	Found in well-defined structures in sea urchins and sea cucumbers.	Exhibits potential effects in anticoagulation, antithrombosis, anti-angiogenesis, antitumor, anti-inflammation and antimicrobial infections [14,15,16].
	SGs	Gal (2S and/or 3S, and/or 4S)	Found in well-defined structures in red algae, sea urchins and ascidians.	

## 3. Medical Mechanisms of Action

### 3.1. Anticoagulant/Antithrombotic Mechanisms of Action

#### 3.1.1. The Serpin-Dependent Mechanisms

Under normal physiological conditions, GAGs on proteoglycans at the endothelial surface play a key role in haemostasis as accelerators of the serpins AT and HCII. These effects lead to enhanced AT and HCII activities of inhibition over certain procoagulant factors such as IIa (thrombin), IXa and Xa [25,38,39,40,41]. The GAG-catalyzed effects on AT and HCII over the blood serine-proteases result from two concomitant molecular events. (i) A template effect by which the sulfated glycans accelerates the formation of the serpin-protease-sulfated glycan ternary complex. In this molecular event the sulfated glycan serves as a ‘molecular bridge’ that will act bringing together both serpin and protease for a close contact. (ii) An allosteric mechanism by which the sulfated glycan induces a conformational change on the serpin in order to achieve a more active structure for interaction and complex formation.

Exogenous sulfated glycans, such as marine SFs and SGs endowed with the capacity of interacting with serpins and their blood targets, will work as synergic molecules in the two molecular events described above for the physiological GAGs. The contribution and quality of these events will depend, of course, on the dose of the exogenous SFs and SGs administered in the system as well as on the presence and concentration per molecule of the active motifs required for interaction with the blood co-(factors) [14,15,16,17,18,19,20,21]. Table 2 shows half maximal inhibitory concentration (IC_50_) values from IIa and Xa inhibition assays via AT and HCII activities using purified enzymes and co-factors as well as the values determined in a coagulometer through the activated partial thromboplastin time (aPTT) method and expressed in units/mg. Values were obtained from curves of anticoagulation time as a function of increasing concentrations of the tested sulfated glycan. A parallel curve generated with commercially available Heparin Sodium sample was obtained as calibrator. In a comparative perspective taking together the anticoagulant potencies (Table 2) and their chemical structures (Figure 2) which enable confident SAR analyses, the structural requirements for the anticoagulant activities of the tested SFs and SGs were discovered. They are the 2-sulfated Gal units as seen in the sea urchin *Echinometra lucunter* SG and the 2,4-di-sulfated Fuc units seen in the SFs from the sea urchin *Strongylocentrotus purpuratus* as well as in the two SGs from the red algal species *Botryocladia occidentalis* and *Gelidium crinale* (Rhodophyta, Rhodymeniophycidae). These structural features have been proposed as the anticoagulant structural motifs of the marine sulfated glycans of well-defined chemical structures [42,43]. Although exhibiting lower anticoagulant activity than heparin, the active compounds do not show the high-bleeding risk of heparin as determined through *in vivo* assays of thrombosis [19,26].

**Table 2:** Anticoagulant activities of marine sulfated glycans of well-defined structures (Figure 2) measured by activated partial thromboplastin time (aPTT) and by IC_50_ values of thrombin (IIa) and factor Xa inhibition in the presence of antithrombin (AT) or heparin cofactor II (HCII).

Polysaccharide Type	Source	aPTT (units/mg) ^a^	IC_50_ (μg/mL)		
			IIa/AT	IIa/HCII	Xa/AT
Invertebrate 3-linked α-l-SF	*S. purpuratus* I	76	0.3	0.3	2
	*S. purpuratus* II	10	0.9	2	nd ^b^
	*S. pallidus*	18	>500	>500	>500
	*L. variegatus* I	3	>500	>500	>500
	*S. franciscanus*	~2	>500	>500	250
	*L. grisea*	<1	>500	>500	>500
Invertebrate 4-linked α-l-SF	*S. droebachiensis*	<1	nd	nd	nd
	*A. lixula*	~2	150	150	>500
Invertebrate α-l-SG	*E. lucunter*	20	3	6	20
	*G. crenularis*	<1	nd	nd	Nd
Red algal SGs	*B. occidentalis*	93	0.02	1.1	2.5
	*G. crinale*	65	0.02	25	1.5

^a^ The activity was measured in units/mg through curves of coagulation time as a function of increasing concentrations of the polysaccharide. A parallel standard curve of an assayed unfractionated heparin sample (commercially available Heparin Sodium) was generated and the activity of this calibrator was measured as 193 units/mg. ^b^ not determined.

#### 3.1.2. The Serpin-Independent Mechanism

**Figure 3:**
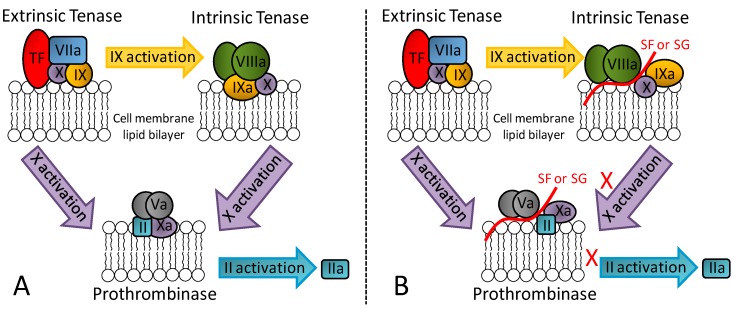
(**A**) Representation of the three key coagulation complexes: extrinsic tenase, intrinsic tenase and prothrombinase. (**B**) The serpin-independent anticoagulant mechanism of marine non-GAG sulfated glycans (SF or SG) relies on the assembly inhibition of the intrinsic tenase and protrombinase complexes.

In addition to its great serpin-dependent anticoagulant activity, it was recently discovered through assays using serpin-free plasma that the SG from the red algae *B. occidentalis* is also able to prolong coagulation time and delay generation course of factors IIa and Xa [17]. In contrast, heparin as the principal anticoagulant GAG of medicine does not exhibit such effects. Further investigation on this unusual anticoagulant effect using purified proteins of the blood coagulation system have led to conclusions that the SG from the red algae *B. occidentalis* is capable of impairing the proper molecular assembly in the intrinsic tenase and prothrombinase complexes. Figure 3 illustrates the physiological (panel A) and impaired (panel B) molecular assemblies. As depicted, these complexes are critical for activation of factors Xa and IIa, respectively. Therefore, an anticoagulant outcome arises from the exogenous application of SG from *B. occidentalis* via an effect unrelated with AT and HCII activities [17].

Recent data have demonstrated that this serpin-independent anticoagulant activity of the SG from red algae can also be seen in *in vivo* models of thrombosis [19]. In fact, through both *in vivo* and *in vitro* assays, the serpin-independent effect comprises the main mechanism of action for the anticoagulant/antithrombotic outcomes of these molecules [18,19]. Results coming from a different research group also concerning the serpin-independent anticoagulant activity of marine sulfated glycans, but specifically on the holothurian GAG named fucosylated chondroitin sulfate, have led to the same conclusion [44]. Hence, although serpin-dependent anticoagulant and antithrombotic SFs and SGs can be measured and structural motifs responsible for such activities can be proposed [41,42], the serpin-unrelated mechanisms dominate in therapy. This conclusion is recent. Future investigations, especially those focused on the right biologically active structural motifs necessary to the serpin-independent mechanisms are worth being carried out.

### 3.2. Anti-Angiogenic and Anticancer Mechanisms of Action

In angiogenesis, the activity of angiogenic, mitogenic, chemotatic and growth-stimulating factors like vascular endothelial growth factor (VEGF) and basic fibroblast growth factor (bFGF, also known as FGF-2) is relevant to the success of the event [45]. These growth factors have to interact and be activated by a resultant ternary complex made up with their canonical receptors and the endothelial surface proteoglycan GAGs [46,47,48,49,50,51,52,53]. Assuming that these factors are circulating free in the plasma, the attachment on surface GAGs is required for their activities in the specific sites of neovascularization. The actions of these growth factors are commonly seen in the cell differentiation event named mesenchymal-epithelial transition, a process which permits the formation of new endothelial cells from angioblasts, and these, in turn, form mesodermal cells [54]. Besides assisting the cell differentiation processes, growth factors also work on the molecular networks involved in the neovascularization [45]. Angiogenesis is a pivotal event in cancer growth (of the primary tumor) and metastasis [55,56]. Formation of new vessels intended to feed the tumor cells are needed for the development of this severe pathology [55,56].

Since binding and attachment of growth factors to endothelial GAGs is required for angiogenesis, administration of certain sulfated glycans in effective concentration will result in a competition process with the functional endothelial GAGs. This could therefore give rise to an anti-angiogenic outcome. Based on this premise, administration in the system of exogenous sulfated glycans with active structural features is likely able to decrease the rates of neovascularization [57,58]. Indeed marine sulfated glycans such as brown algal fucoidans display competitive structural and functional properties against endothelial GAGs and are considered great anti-angiogenic and anticancer sulfated polysaccharides. Both *in vitro* and *in vivo* experiments have been performed to evaluate the anti-angiogenic and anticancer potentials of these glycans [57,58,59].

Although useful in advanced SAR studies, data regarding the marine SFs and SGs of well-defined chemical structures (Figure 2) as anti-angiogenic and antitumor agents are virtually inexistent up to now. On the other hand, since the brown algal fucoidans have been preferred to the investigations related to these systems, some SAR information concerning fucoidans has been proposed. It seems that molecular size of fucoidans is an important feature in anti-angiogenesis [58]. Based on what has been highlighted by Prof. Boisson-Vidal [60] and supported by the recent analyses of Ustyuzhanina and associates [58], high-molecular weight fucoidans (usually above 30 kDa) bearing also high degrees of sulfation, are likely able to present anti-angiogenic affects. Conversely, low-molecular weight fucoidans (usually below 15 kDa) or fucoidan oligosaccharide fractions tend to promote angiogenesis. This molecular weight-dependent antagonic effects in angiogenesis of the brown algal fucoidan must be further investigated for anticancer therapy.

### 3.3. Anti-Inflammatory Mechanisms of Action

**Figure 4:**
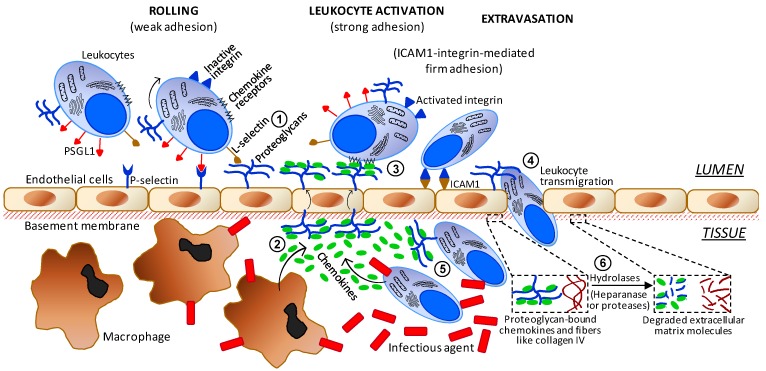
Simplified scheme regarding the major cells and molecular players involved in the progress of inflammation. The numbers indicate the sites of action of the anti-inflammatory sulfated glycans. Abbreviations used are TNF for tumor necrosis factor, ICAM for intercellular adhesion molecule and PSGL1 for P-selectin glycoprotein ligand-1.

It is well-established that carbohydrates including sulfated glycans play key roles in events of cell–cell and cell–matrix communication. These molecules can either promote or inhibit the steps of inflammation. In addition, this category of molecules is attracting much attention nowadays in research programs involved with the molecular details and therapeutic agents of inflammation [61,62,63]. Amongst all glycans reported to be active in inflammation, surface GAGs of both circulating blood cells and endothelial proteoglycans are those with the highest number of roles. As illustrated in Figure 4, they are involved in (1) interactions with L-selectin during the initial steps of leukocyte recruitment and rolling; (2) interactions and storage of released chemokines from macrophages in the underlying tissue; (3) after translocation of the proteoglycan-retained chemokines facing the inner tissue, these peptides are presented to their respective leukocyte receptors in order to trigger leukocyte activation; (4) facilitation on the adequate extravasation of activated leukocytes through the tight spaces of the endothelial barrier; (5) after activated leukocytes have gained access into the inflamed inner tissue, the infiltrated leukocytes will interact with the yet-unbound chemokines available at that region via surface proteoglycan GAGs; and (6) in order to promote a pro-inflammatory upstream in the system, leukocytes that have entered into the inner tissue become able to release hydrolases capable of degrading proteoglycans and collagen fibers of the ECM close to the underlying basement membrane. This later process is important to facilitate leukocyte transmigration through the endothelial layer.

Since multiple roles can be assigned to GAGs in inflammation progress; it wouldn't be unexpected if exogenous sulfated glycans could be used as possible therapeutic anti-inflammatory agents. This outcome arises from the fact that when sulfated glycans are administered in effective concentrations in cases of acute or chronic inflammatory disorders, these molecules are likely able to compete with the physiological GAGs needed to keep the proper molecular workflow of this pathophysiological event. Hence, sulfated glycans from diverse sources and various structures such as the marine SFs and SGs are currently under investigation as potential anti-inflammatory agents [57,64,65,66,67,68]. It has been postulated that the anti-inflammatory activities of these marine sulfated glycans depend directly on the structure of the polysaccharide used for treatment [57,64,65,66,67,68].

### 3.4. Antimicrobial Mechanism of Action

**Figure 5:**
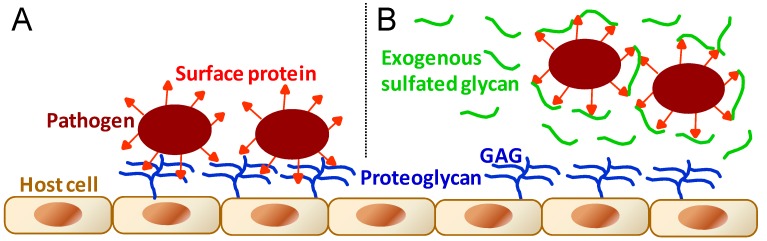
General schematic representation for the molecular mechanisms during (**A**) the microbial infection of pathogens such as bacteria, virus, fungus, and protozoan parasites; and during (**B**) the administration at certain concentrations of exogenous sulfated glycans endowed with antimicrobial activity. In (**A**), microbial infection is driven by molecular interactions between pathogen proteins and host cell GAGs. In (**B**), the antimicrobial sulfated glycan competes with host cell GAGs by binding to proteins displayed at the microbial surface, impairing or disrupting then the pathogen attachment onto host cells.

As stated in the beginning of the previous section, GAGs are essential molecules to events involving cell–cell interactions. This can be easily exemplified by interactions of microbial pathogens with their target cells in host organisms as seen in the initial stages of microbial infectivity of mammalian cells. GAGs, as the main physiological sulfate glycan representatives of host cells, are one of the principal molecules involved in attachment, adhesion and entry of the various types of micropathogens (Figure 5A) including bacteria [69,70,71,72,73,74], virus [69,70,74,75,76,77,78,79], fungus [69,70,80], and protozoan parasites [69,70,81,82].

Based on the molecular mechanisms illustrated in Figure 5, sulfated glycans including commercially available GAGs and marine SFs and SGs are clearly able to result in an antimicrobial outcome when applied in events characterized by micropathogen infectivity [83,84,85,86,87,88,89]. The antimicrobial activity of sulfated glycans arises from their capacity to compete with the host cell GAGs necessary to the progress of the microbial recognition, attachment and invasion (Figure 5B). The levels of response for the antimicrobial treatment will depend, of course, on the dose of the active glycan administered in the system as well as on the structural features of the chosen sulfated glycans. The marine sulfated glycans most investigated so far concerning their antimicrobial activities have been the brown algal fucoidans [86,87] and the red algal carrageenans [87,88,89], a type of SG composed of disaccharide repeating units and usually regular patterns of sulfation. No firm SAR conclusions about these red and brown algal sulfated glycans have been proposed so far as new antimicrobial agents.

## 4. Concluding Remarks and Existing Challenges in Drug Development

Sulfated glycans are perhaps the carbohydrate types most studied in what concerns their medical activities. Mammalian-derived GAGs such as heparin, chondroitin sulfate and keratan sulfate are examples of these well-known medical sulfated glycans. Non-GAGs such as marine sulfated glycans are another type of sulfated glycans also endowed with medical properties. SFs and SGs are the main examples of these marine polysaccharides. They are available to be used as alternative or supplement agents in GAG-based therapies. Table 3 highlights in a comparative fashion the effects of the therapeutic GAGs and the marine SFs and SGs in the series of pathophysiological systems discussed in this document.

**Table 3:** Medical effects of exogenous GAGs and marine sulfated glycans of well-defined chemical structures in different pathophysiological systems.

Medical System	GAGs	Marine Sulfated Glycans
Anticoagulation and antitrombosis	Serpin-dependent mechanism - Accelerate AT inhibition over factors IIa and Xa. - Accelarate HCII inhibition over factor IIa.	Serpin-dependent mechanism - Accelerate AT inhibition over factors IIa and Xa. - Accelarate HCII inhibition over factor IIa. Serpin-independent mechanism - Inhibit intrinsic tenase complex. - Inhibit prothrombinase complex.
Anti-angiogenesis and anticancer	- Inhibition activities on growth factors necessary for cell differentiation and neovascularization. - Inhibition activities on selectins necessary for cell migration, attachment and adhesion.	
Anti-inflammation	- Inhibit L-selectin during leukocyte recruitment and rolling. - Inhibit chemokine functions in leukocyte activation. - Decrease extravasation of activated leukocytes. - Inhibit binding of infiltrated leukocytes to free chemokines in the inflamed tissue. - Compete with hydrolase activity during ECM processing necessary to enhance leukocyte transmigration. Marine sulfated glycans maybe less active or inactive in this effect.	
Antimicrobial infections	- Compete with host cell GAGs during host cell-pathogen recognition, interaction and attachment during microbial infection.	

As detailed above, SFs and GSs of well-defined chemical structures are promising drug candidates because they exhibit a phenomenal number of medical benefits in multiple pathophysiological systems. Conversely, the real clinical application of these marine sulfated glycans is still some years away. Although studies of clinical trials using patients committed with chronic or acute disorders involving the pathologies which the marine sulfated glycans are known to be active and have already been approved and initiated [59,90], fucoidans seem to be the only representative molecules of marine sulfated glycans under current trials. As detailed here, these molecules are heterogeneous and accurate SAR conclusions regarding their structural features are hard to be proposed. In addition to this, some marine sulfated glycans can respond in a similar way to the oversulfated chondroitin sulfate in respect to the capacity of activating coagulation blood factor XII [19,91]. Factor XIIa can participate in inducing severe hypotension caused by kallikrein, a potent regulator of blood pressure via activation of bradykinin. This can lead to a harmful effect to health during a possible therapy of the marine sulfated glycans equipped with kallikrein-dependent effects. This downside of certain marine sulfated glycans is a serious challenge for the future implementation of these compounds in the next generation of potential carbohydrate-based drug candidates. Nonetheless, interest and efforts in research regarding the medicinal benefits of these marine sulfated glycans are growing considerably in the last few years across scientific groups of different countries. We expect some day that the amount of information concerning the beneficial and the harmful effects to health of the marine sulfated glycans reach a reliable point in order to make these molecules much more accessible to patients suffering from the pathologies they are active.
